# Comparative assessment of standalone and hybrid deep neural networks for modeling daily pan evaporation in a semi-arid environment

**DOI:** 10.1038/s41598-025-05985-z

**Published:** 2025-06-20

**Authors:** Mohammed Achite, Manish Kumar, Nehal Elshaboury, Aman Srivastava, Ahmed Elbeltagi, Ali Salem

**Affiliations:** 1 Faculty of Nature and Life Sciences, Water and Environment Laboratory, University Hassiba Benboual of Chlef, Chlef, B.P. 78C, Ouled Fares, 02180 Algeria; 2 College of Agricultural Engineering and Technology, Dr. R.P.C.A.U, Pusa, Samastipur, Bihar 848125 India; 3 Construction and Project Management Research Institute, Housing and Building National Research Centre, Giza, 12311 Egypt; 4 Formerly, Centre for Technology Alternatives for Rural Areas (CTARA), Indian Institute of Technology (IIT) Bombay, Mumbai, 400076 India; 5 Agricultural Engineering Department, Faculty of Agriculture, Mansoura University, Mansoura, 35516 Egypt; 6 Civil Engineering Department, Faculty of Engineering, Minia University, Minia, 61111 Egypt; 7 Structural Diagnostics and Analysis Research Group, Faculty of Engineering and Information Technology, University of Pécs, Pecs, Hungary

**Keywords:** Pan evaporation, Meteorological variables, Machine learning, Deep neural network, Semi-arid regions, Environmental sciences, Hydrology

## Abstract

Evaporation represents a fundamental hydrological cycle process that demands dependable methods to quantify its fluctuation to ascertain sustainable agriculture, irrigation systems, and overall water resource management. Meteorological variables such as relative humidity, temperature, wind speed, and sunshine hours affect evaporation non-linearly, resulting in challenges while developing prediction models. To combat this, the study aimed to develop robust models for estimating evaporation in semi-arid environments by applying machine learning techniques. Daily meteorological datasets (from January 2000 to December 2010) for the above variables (input) were collected from the Sidi Yakoub meteorological station in the Wadi Sly basin, Algeria. Conventional deep neural network (DNN) coupled with support vector machine (SVM), Bayesian additive regression trees (BART), random subspace (RSS), M5 pruned, and random forest (RF) were used for developing prediction models using various input variable combinations. Model performances were compared using mean absolute error (MAE), root mean square error (RMSE), determination coefficient (R^2^), Nash–Sutcliffe efficiency (NSE) coefficient, and percentage bias (PBIAS). Results indicated comparatively better performance for hybrid models (DNN-SVM, DNN-BART, DNN-RSS, DNN-M5 pruned, and DNN-RF) than conventional models (standalone DNN). Among hybrid models, the DNN-SVM model outperformed others with high accuracy and performance and fewer statistical errors in the daily pan evaporation prediction during the testing phase (R²=0.65, RMSE = 3.00 mm, MAE = 2.13, NSE = 0.65, and PBIAS = 3.54). DNN-RF was in the second rank for the prediction with R^2^ of 0.64, RMSE of 3.00 mm, MAE of 2.16, NSE of 0.64, and PBIAS = 0.41. While the standalone DNN model gave the lowest results with MAE of 4.87, RMSE of 5.00 mm, and NRMSE of 0.65. The present framework’s success in Algeria’s Wadi Sly basin highlights its potential for scalable adoption in irrigation scheduling and drought resilience strategies, yielding implementable steps for policymakers, addressing climate-driven water scarcity. Future research should explore integrating real-time climate projections and socio-hydrological variables to improve predictive adaptability across diverse agroecological zones.

**Supplementary Information:**

The online version contains supplementary material available at 10.1038/s41598-025-05985-z.

## Introduction

Evaporation losses have significant global implications, particularly in the context of climate change, human interventions, and natural climate oscillations^1^. Rising global temperatures have intensified evaporation rates, exacerbating water scarcity issues in many regions, especially arid and semi-arid areas^2^. Increased evaporation from reservoirs, lakes, and river basins threatens freshwater availability, impacting agriculture, drinking water supplies, and hydropower generation^3^. Additionally, anthropogenic activities such as land-use changes, deforestation, and urbanization alter local climate patterns, further influencing evaporation rates and disrupting regional water cycles^4^. Climate oscillations, such as El Niño and La Niña, also play a crucial role in modifying evaporation patterns, leading to extreme droughts or intense precipitation events that impact water storage and distribution^5^. Given these challenges, accurately quantifying evaporation losses is essential for sustainable water resource management, agricultural planning, and climate adaptation strategies^6^.

Evaporation is measured using either direct methods, such as the pan evaporation method, or indirect methods, such as semi-empirical and empirical models^7^. Pan evaporation is commonly used for measuring evaporation because of its simplicity and cost-effectiveness^8,9^. The Class A pan is the most widely used method of assessing surface evaporation because it allows researchers to compare the evaporation rates in different locations^10^. However, it cannot be deployed everywhere, especially in difficult areas where instrumentation cannot be installed or maintained^11^. Therefore, the implementation cost is a disadvantage in developing nations^12^. As a result, studies have suggested developing indirect evaporation estimation methods (i.e., empirical and semi-empirical models) from various metrological parameters, such as mean temperature (T_mean_, ℃), maximum temperature (T_max_, ℃), minimum temperature (T_min_, ℃), wind speed (WSP, ms^− 1^), solar radiation (R_s_, MJm^2^), extra-terrestrial radiation (R_a_, MJm^2^), precipitation (P, mm), sunshine hours (SSH, hours), vapor pressure (VP, hPa), rainfall (R, mm), and relative humidity (RH, %)^13^. Penman-Monteith, Thornthwaite, and Priestley-Taylor equations are examples of empirical models^14^. However, due to the stochasticity of the meteorological variables and the location-specific nature of these models, the empirical approaches might underestimate or overestimate pan evaporation (Ep) under a wide range of climatic conditions, particularly during extreme weather events^15^. Hence developing robust machine learning (ML) models for predicting evaporation becomes an imperative approach^16–18^.

Over the last two decades, ML models have made significant improvements in several hydrological and climatological fields, including drought^19,20^, rainfall^21,22^, evapotranspiration^23,24^, surface water quality^25^, and streamflow^26,27^. This is owing to the capacity of these models to handle complex and stochastic problems^28^. A survey of related articles is conducted to grasp an overview of the most recent work in this field, as depicted in Table 1.

After reviewing the above-mentioned research studies, it has been recognized that ML models have been widely applied to estimate Ep across various regions worldwide. Compared to traditional empirical methods, ML-based approaches demonstrate superior predictive accuracy due to their ability to capture complex, nonlinear relationships between meteorological parameters^29^. The evolution of ML models in earth and atmospheric sciences has seen a transition from conventional statistical and empirical methods to more sophisticated deep learning frameworks. Early studies primarily relied on artificial neural network (ANN) and support vector machine (SVM) for hydrological modeling, benefiting from their ability to learn from data without explicit physical assumptions^30^. However, these models often required extensive parameter tuning and were limited in their capacity to handle high-dimensional datasets effectively. Recent advancements in deep learning have introduced more powerful architectures, such as deep neural networks (DNNs) and hybrid models, which integrate multiple ML techniques for improved accuracy and generalization^31^. Studies have shown that hybrid approaches, such as combining DNN with SVM or random forest (RF), can enhance predictive performance by leveraging the strengths of multiple algorithms^32^. Additionally, ensemble learning techniques and Bayesian frameworks, such as Bayesian additive regression trees (BART), have gained traction in atmospheric and hydrological studies due to their robustness in handling uncertainties and complex spatial-temporal patterns.

Despite significant advancements in ML applications for hydrological modeling, the generalization capability of ML models across diverse climatic regions remains a challenge due to the stochastic and region-specific nature of meteorological conditions. To address this limitation, the present study evaluates six ML models—conventional DNN, DNN coupled with SVM, BART, random subspace (RSS), M5 pruned trees, and RF—for predicting daily pan evaporation rates in semi-arid regions. The study utilizes daily meteorological data from the Sidi Yakoub meteorological station in the Wadi Sly basin, Algeria, incorporating key climatic variables such as sunshine hours, wind speed, and relative humidity and temperature (mean, maximum, and minimum). The novelty of this research lies in its comparative assessment of hybrid and ensemble ML models, integrating advanced techniques such as DNN-SVM and ensemble learning methods like BART and RF to enhance predictive accuracy. Unlike previous studies that primarily focus on standalone models, this study systematically evaluates multiple approaches to improve evaporation estimation. Additionally, it provides the first application of these models in Algeria’s semi-arid regions, addressing a critical gap in hydrological modeling for arid and semi-arid climates. The research also employs sensitivity analysis to optimize input variable selection, ensuring that the most influential meteorological parameters are identified for precise prediction. Finally, a rigorous performance evaluation using multiple statistical metrics strengthens the reliability of the findings, contributing valuable insights for sustainable water resource management in regions with high evaporation rates. By addressing these aspects, this study contributes to improving the reliability and adaptability of ML-based evaporation prediction models, offering valuable insights for sustainable water resource management in semi-arid regions.

**Table 1:** Summary of research studies for modeling and estimating evaporation in different regions.

Reference	Applied models	Study location	Climatic inputs	Evaluation metrics	Recommended model
Adnan et al.^33^	Clustered ANFIS using: Grid partition Subtractive clustering FCM	Uttarakhand station, India	T_min_, T_max,_ WSP, SSH, and RH	R^2^, NSE, RMSE, and MAE	ANFIS-FCM
Ghaemi et al.^34^	MODWT coupled with: MARS M5 model tree	Siirt and Diyarbakir stations, Turkey	T, WSP, RH, R_s_	RMSE, NSE, MAE, and R	MARS^MODWT^
Khosravi et al.^35^	Data mining models: M5 pruning REPT RF Random tree Kstar Hybrid models: ANFIS ANFIS-differential evolution algorithm ANFIS-GA ANFIS-imperialistic competitive algorithm	Baghdad and Mosul stations, Iraq	WSP, SSH, P, RH, T_min_, and T_max_	R^2^, NSE, RMSE, MAE, RSR, and PBIAS	ANFIS-GA
Kisi and Heddam^36^	MARS M5 model tree	Adana, Antakya and Mersin stations, Turkey	T_min_, T_max_, and R_a_	RMSE, MAE, and NSE	MARS
Rezaie-Balf et al.^37^	EEMD coupled with: SVM M5 model tree	Siirt and Diyarbakir stations, Turkey	WSP, T, RH, and R_a_	NSE, RMSE, PI, WI, and LMI	EEMD-M5 model tree
Sebbar et al.^38^	OSELM OPELM	Ain Dalia and Zit Emba stations, Algeria	T_min_, T_max_, WSP, and RH	RMSE, MAE, and R	OPELM (Ain Dalia) and OSELM (Zit Emba)
Alsumaiei^39^	ANN trained using different meteorological variable combinations	Kuwait International Airport, Saberya, and Abdaly stations, Kuwait	T_mean_, WSP, and RH	R, R^2^, MAE, and NSE	ANN
Malik et al.^3^	MM-ANN MARS SVM MGGP M5 model tree	Ranichauri and Pantnagar stations, India	T_min_, T_max_ WSP, SSH, and RH	LMI, WI, RMSE, NSE, and MAPE	MM-ANN and MGGP
Shabani et al.^6^	GPR KNN SVR RF	Gonbad-e Kavus, Gorgan, and Bandar Torkman stations, Iran	T, RH, WSP, and SSH	RMSE, R, and MAE	GPR
Wu et al.^9^	ELM coupled with: Whale optimization algorithm FPA	Poyang Lake Basin, Southern China	T_min_, T_max,_ SSH, RH, and WSP	RMSE, MAE, MAPE, R^2^, and NSE	FPA-ELM
Yaseen et al.^40^	Cascade correlation neural network Classification and regression tree SVM GEP	Baghdad and Mosul stations, Iraq	T_min_, T_max_, P, RH, and SSH	MAE, RMSE, NSE, WI, LMI, and R^2^	SVM
Al-Mukhtar^14^	Conditional random forest regression MARS Bagged MARS M5 model tree KNN Weighted KNN	Baghdad, Basrah, and Mosul stations, Iraq	T_min_, T_max_, T_mean,_ WSP, and RH	R^2^, NSE, MAE, RMSE, and PBIAS	Weighted KNN
Abed et al.^41^	RF CNN DNN	Bayan Lepas, Ipoh, KLIA Sepang, and Kuantan, Malaysia	T_max_, T_min_, T_mean_, RH, WSP, and R_s_	R^2^, MAE, MSE, RMSE, RAE, and NSE	CNN
Ehteram et al.^42^	Optimized KELM using: BMA WSA Salp swarm algorithm Shark algorithm Particle swarm optimization	Hormozgan, Mazandaran, Fars, Yazd, and Isfahan provinces, Iran	T_mean_, SSH, WSP, RH, and R	RMSE, MAE, PBIAS, p, and w	BMA and KELM-WSA
Novotná et al.^43^	ANN DT Autoneural network Dmine regression DM neural network Gradient boosting Least angle regression Ensemble model	Slovak Republic	T_max_, T_min_, and T_mean_, RH, WSP, R, VP, elevation, coordinates, and wind directions	Average squared error	Dmine regression
El Bilali et al.^44^	DNN SVR Extra tree XGBoost	Sidi Mohammed Ben Abdellah, Morocco	T_mean_, WSP, RH, VP, and R_s_	NSE, RMSE, and PBIAS	XGBoost
Elbeltagi et al.^45^	AR coupled with: RSS M5 pruned REPT Bagging	Baghdad, Mosul, and Basrah, Iraq	T_max_, T_min_, T_mean_, RH, and WSP	MAE, RMSE, RAE, RRSE, and R	AR-M5 pruned
Fu and Li^46^	LSTM and SVM optimized using: GWO Whale optimization algorithm	Shapotou, Ningxia Hui Autonomous Region, China	T_max_, T_min_, T_mean_, VP, and WSP	RMSE, NMSE, MAE, MAPE, and NSE	LSTM-GWO
Mohammed et al.^47^	MLR	Ramadi City, Iraq	T_max_, T_min_, T_mean_, WSP, RH, and R_s_	RMSE, NAE, MAPE, NSE, and R^2^	MLR
Rajput et al.^48^	Bagging RSS M5 pruned REPT	New Delhi, India	T_mean_	R, MAE, RMSE, RAE, RRSE, MBE, NSE, WI, KGE, and MAPE	Bagging

Models: *ANFIS* Adaptive neuro-fuzzy inference system, *ANN* Artificial neural network, *AR* Additive regression, *BMA* Bayesian model averaging, *CNN* Convolutional neural network, *DNN* Deep neural network, *DT* Decision tree, *EEMD* Ensemble empirical mode decomposition, *ELM* Extreme learning machine, *FCM* Fuzzy c-means clustering, *FPA* Flower pollination algorithm, *GA* Genetic algorithm, *GEP* Gene expression programming, *GPR* Gaussian process regression, *GWO* Grey wolf optimizer, *KELM* Kernel extreme learning machine, *KNN* K-nearest neighbor, *LSTM* Long short-term memory, *MARS* Multivariate adaptive regression spline, *MGGP* Multi-gene genetic programming, *MLR* Multiple linear regression, *MM-ANN* Multiple model-ANN, *MODWT* Maximum overlap discrete wavelet transform, *OPELM* Optimally pruned extreme learning machine, *OSELM* Online sequential extreme learning machine, *REPT* Reduced error pruning tree, *RF* Random forest, *RSS* Random subspace, *SVM* Support vector machine, *SVR* Support vector regression, *WSA* Water strider algorithm, *XGBoost* Extreme gradient boosting.

Performance metrics *KGE* Kling-Gupta efficiency, *LMI* Legates–McCabe’s index, *MAE* Mean absolute error, *MAPE* Mean absolute percentage error, *MBE* Mean bias error, *NAE* Normalized absolute error, *NMSE* Normalized mean squared error, *NSE* Nash-Sutcliffe efficiency, *p* Percentage of measured data bracketed by 95% of predicted uncertainties, *PBIAS* Percentage of bias, *PI* Performance index, *R* Correlation coefficient, *R*^2^ Determination coefficient, *RAE* Relative absolute error, *RMSE* Root mean square error, *RRSE* Root relative square error, *RSR* Ratio of RMSE to the standard deviation of observations, *w* Width of uncertainty bound, *WI* Willmott’s index.

## Materials and methods

### Study area and data collection

**Fig. 1:**
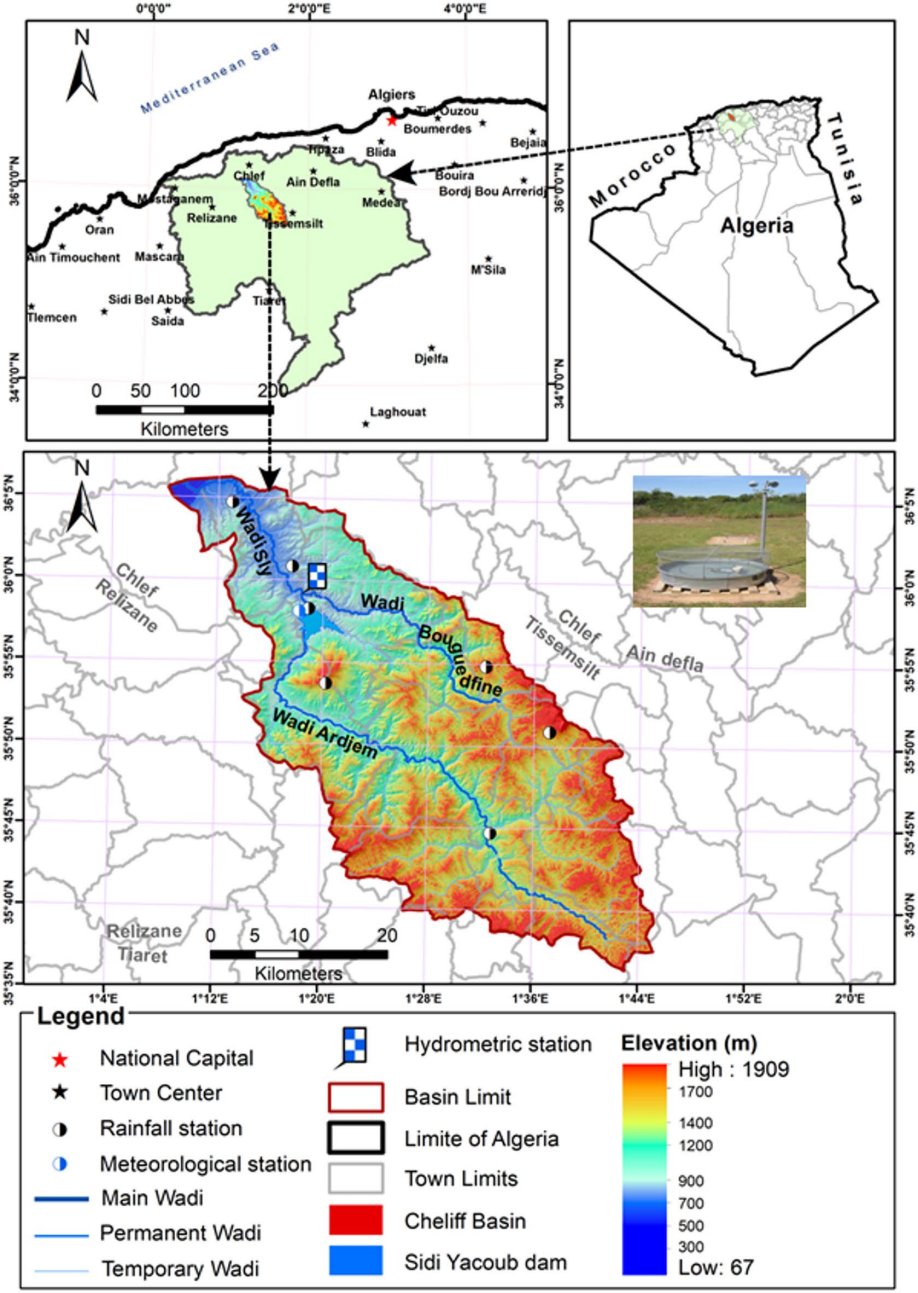
Location of the study area and the meteorological station (created by ArcGIS 10.8.2).

As shown in Fig. 1, the study area is the Wadi Sly basin, located in northwest Algeria. It has an area of 1,400 km^2^ with coordinates of 35° 36’ 5”–36° 5’ 53” N and 1° 8’ 16”–1° 44’ 56” E. The basin has a maximum width and length of 30 and 70 km, respectively. Besides, it has a narrow, long-form, and large hydrographic network. The Sidi Yakoub dam, built for agricultural purposes, influences flows in the lower section of the basin. According to the Köppen-Geiger classification, the basin’s climate is the Mediterranean, with extremely hot summers and moderate winters. The coldest month is often January, whose temperature is typically above 0 °C. Besides, the average temperatures of at least one and four months are above 22 °C and 10 °C, respectively. In this environment, rainfall in the wettest month of the year is generally three times that of the driest month of the year.

The Wadi Sly basin in northwest Algeria was chosen as the study area for several reasons. Firstly, it represents a typical semi-arid environment with a Mediterranean climate, characterized by hot summers and mild winters, making it an ideal location to study evaporation. The basin’s hydrological characteristics, including its extensive hydrographic network and the presence of the Sidi Yakoub dam, provide a complex yet controlled environment to assess the impact of meteorological variables on evaporation. Additionally, the region’s reliance on irrigation for agriculture underscores the importance of accurate evaporation modeling for sustainable water resource management. The availability of comprehensive meteorological data from the Sidi Yakoub meteorological station further facilitated the selection of this site for the study.

The daily meteorological data, including minimum, maximum, and mean air temperature (T_min_, T_max,_ and T_mean_), minimum, maximum, and mean relative humidity (RH_min_, RH_max,_ and RH_mean_), wind speed (WSP), sunshine hours (SSH), and pan evaporation (Ep) are collected from the Sidi Yakoub meteorological station. The dataset comprises daily records for 11 years from January 2000 to December 2010 (Figure S1). Table 2 shows the statistical characteristics of the meteorological data.

**Table 2:** Statistical measures of the meteorological data for training and testing sets.

Statistical parameter	T_min_ (°C)	T_max_ (°C)	T_mean_ (°C)	RH_min_ (%)	RH_max_ (%)	RH_mean_ (%)	SSH (h)	WSP (m s^− 1^)	Ep (mm)
All data (4018)									
Minimum	0.10	6.30	3.35	2.50	33.00	30.00	0.00	0.00	0.10
Maximum	33.30	47.40	40.15	87.50	99.00	89.50	14.20	11.40	23.00
Mean	14.57	25.82	20.19	36.03	71.83	53.93	8.00	2.83	7.62
Standard deviation	6.27	9.05	7.49	17.42	12.23	13.88	3.79	1.41	5.11
Kurtosis	− 0.89	− 1.08	− 1.05	− 0.77	− 0.50	− 0.95	− 0.67	2.93	− 0.82
Skewness coefficient	0.18	0.18	0.20	0.33	− 0.62	0.00	− 0.58	1.19	0.48
Training phase (3000)									
Minimum	0.10	6.30	3.35	2.50	33.00	30.00	0.00	0.00	0.10
Maximum	33.30	47.10	40.15	87.50	99.00	89.50	14.20	9.90	23.00
Mean	14.57	25.81	20.19	35.49	70.91	53.20	7.92	2.73	7.62
Standard deviation	6.44	9.15	7.63	17.44	12.28	13.94	3.75	1.25	5.08
Kurtosis	41.41	83.81	58.19	304.18	150.79	194.23	14.08	1.57	25.79
Skewness coefficient	− 0.95	− 1.14	− 1.11	− 0.73	− 0.59	− 0.94	− 0.64	1.91	− 0.77
Testing phase (1018)									
Minimum	1.40	7.20	5.05	2.50	42.50	30.00	0.00	0.00	0.10
Maximum	30.70	47.40	38.85	85.00	98.00	87.75	13.90	11.40	22.10
Mean	14.55	25.83	20.19	37.62	74.54	56.08	8.24	3.11	7.64
Standard deviation	5.77	8.74	7.08	17.27	11.68	13.48	3.88	1.75	5.20
Kurtosis	− 0.74	− 0.87	− 0.83	− 0.85	− 0.14	− 0.92	− 0.72	2.18	− 0.97
Skewness coefficient	0.24	0.27	0.29	0.23	− 0.80	− 0.14	− 0.63	1.23	0.41

### Machine learning models

For modeling evaporation in Algeria, the present study applies six hybrid ML models; conventional DNN, DNN-SVM, DNN-BART, DNN-RSS, DNN-M5 pruned, and DNN-RF. The process of stacking has been adopted for the hybridization of the models. Stacking is a way to ensemble multiple regression models. It is a general procedure where a learner is trained to combine the individual learners. Here, the individual learners are called the first-level learners, while the combiner is called the second-level learner, or meta-learner. The division of data into training and testing sets in the developed ML models was 75:25. It implies that 75% of the data is used for training the model, while 25% is reserved for testing and evaluating the model’s performance. The architecture of each model is described in the following sub-sections.

#### Deep neural network (DNN)

Hinton et al.^49^ proposed the concept of deep learning and networks that subsequently rejuvenated DNN. The DNN has emerged as a central and powerful deep learning model in the ongoing decade, given its added advantage over the single-hidden-layer ANN model^50,51^. The DNN models provide scope to learn complex non-linear relationships via multiple hidden layers considering features, targets, inputs, and outputs. These multiple layers allow models to understand complex features more efficiently and perform more intensive computational operations. The higher efficiency and computation ability of DNN are due to deep learning algorithms’ ability to learn from their own errors, such that DNN can validate the model prediction accuracy. On the other hand, classical ML models require varying degrees of human intervention to determine output accuracy. Therefore, the DNN model became the natural choice for evaporation estimates and predictions for the present investigation. In this study, a 4-layer DNN model with a rectified linear unit ($\:ReLu$) activation function is employed, as shown in Eq. 1. For this DNN architecture, the loss function ($\:Loss$) is estimated using Eqs. 2–3. In order to minimize the loss, a classical gradient-descent method is applied. TensorFlow software has been used to write the codes of the DNN model in Python 3.6.

1 $$\:ReLu=\left\{\begin{array}{c}x,if\:x>0\\\:0,\:if\:x\le\:0\end{array}\right.$$

2 $$\:Loss=\frac{1}{2n}{\sum\:}_{i=1}^{n}{({EP}_{0}-{Ep}_{DNN})}^{2}\:\:\:\:\:\:$$

3 $$\:{Ep}_{DNN}={w}_{5}\left(ReLu\left({w}_{4}\left(ReLu\left({w}_{3}\left(ReLu\left({w}_{2}\left(ReLu\left({w}_{1}x+{b}_{1}\right)\right)+{b}_{2}\right)\right)+{b}_{3}\right)\right)+{b}_{4}\right)\right)+{b}_{5\:}\:\:)$$.

where $\:n$ is the number of observations or data records, $\:{EP}_{0}$ and $\:{Ep}_{DNN}$ are observed and predicted evaporation by the DNN model, respectively. Besides, in the networks, weights are represented as $\:{w}_{1}$, $\:{w}_{2}$, $\:{w}_{3}$, $\:{w}_{4}$, and $\:{w}_{5}$, while bias terms are represented as $\:{b}_{1}$, $\:{b}_{2}$, $\:{b}_{3}$, $\:{b}_{4}$, and $\:{b}_{5}$.

#### Support vector machine (SVM)

Cortes and Vapnik^52^ established the kernel-based SVM model, a supervised soft computing technique proficient in reducing complexities alongside errors in the estimation. The classifier models of SVMs are applied to data classification problems under different classes. Another group of the SVM is support vector regression, which is used in regression prediction problems. These regression functions are approximate as shown in Eq. 4. Here, the kernel function (input vector $\:x$) implicitly transforms the inputs of the lower-dimensional to a higher-dimensional feature [$\:\beta\:\left(x\right)$], wherein $\:w$ is the weighting vector, and $\:b$ is a bias. These two parameters are estimated using a regularized risk function [$\:R\left(P\right)$], as shown in Eqs. 5, 6.

4 $$\:f\left(x\right)=w\beta\:\left(x\right)+b\:\:\:\:\:\:$$

5 $$\:R\left(P\right)=P\frac{1}{n}{\sum\:}_{i=1}^{n}L({d}_{i},{y}_{i})+\frac{1}{2}{\left|\left|w\right|\right|}^{2}\:\:\:\:\:\:$$

6 $$\:{L}_{\epsilon\:}\left(d,y\right)=\left\{\begin{array}{c}\left|d-y\right|-\epsilon\:\left|d-y\right|\ge\:\epsilon\:\:\:\:\:\:\:\\\:0,\:\:\:otherwise\end{array}\right.$$.

where $\:P$ is a penalty parameter, $\:\frac{1}{2}{\left|\left|w\right|\right|}^{2}$ is a regularization term, $\:{d}_{i}$ is the desired value, $\:P\frac{1}{n}{\sum\:}_{i=1}^{n}L({d}_{i},{y}_{i})$ is the error term, and $\:Ɛ$ is the tube size of SVM in $\:{L}_{\epsilon\:}$. Since one of the advantages of employing SVM is finding a hyperplane in an N-dimensional space that separately classifies the data points, it works comparably well when there is an understandable margin of dissociation between classes, as observed for variables influencing evaporation processes. Furthermore, as the present study employs SVM in a higher dimension such that the target class is not much overlapping and data size is appropriate, SVM functions more productive and effective, becoming a confident choice for predicting evaporation.

#### Bayesian additive regression tree (BART)

In recent years, BART has gained popularity among the research community due to their widespread applications^53,54^. BART models have overcome one of the limitations of ML methods - the lack of uncertainty quantification for individual predictions. In a regression framework, BART quantifies uncertainty through a sum-of-trees approach, in that many decision trees contribute while predicting using a probabilistic model-based method. To execute, BART employs prediction standard error and prediction intervals, and thus it can be proclaimed that techniques like BART are highly appropriate when predicting evaporation where uncertainty is inherent. This study applied BART for additive regression using MATLAB. It has a continuous outcome for pan evaporation (say $\:y$) and $\:p$ covariates $\:x\:=\:({x}_{1},\dots\:,\:{x}_{p})$. The BART prediction model can define complex relations between the aforesaid $\:x$ and $\:y$ by estimating $\:f\left(x\right)$ as follows: $\:y\:=\:f\:\left(x\right)\:+\:\epsilon\:$, where $\:\epsilon\:\:\sim\:\:N(0,\:{\sigma\:}^{2})$. Furthermore, a sum of $\:m$ regression trees is used, i.e., $\:f\left(x\right)\:=\:\sum\:\:g\:(x;\:{T}_{j},\:{M}_{j})$ ranging between $\:j\:=\:1 \sim m$ allows estimation of $\:f\left(x\right)$. The expression for BART is shown in Eq. 7.

7 $$\:y=f\left(x\right)+\epsilon\:={\sum\:}_{j=1}^{m}g(x;{T}_{j},{M}_{j})+\epsilon\:\:\:\:\:\:$$.

#### Random subspace (RSS)

Ho^55^ introduced the RSS ensemble, which is proficient in combining multiple classifiers and their outputs (predictions) from multiple decision trees (DTs) via a voting approach. It overcomes one of the grave shortcomings of conventional DTs^56^. This has been achieved by addressing the overfitting issue of the decision-making tree classifier (i.e., high variance and low bias). Furthermore, it ensures the precision of the training results. Skurichina and Duin^57^ classified inputs of the RSS algorithm into four categories: training dataset, base classifier as well as size and number of subspaces. In the ensemble, the subset of input features (columns) is randomly selected for each model in the first step. While in the second step, the model is attempted to fit during the entire training dataset. To achieve this, bootstraps or random samples (rows) are implemented in the training dataset. RSS generally attempts to reduce the correlation between estimators in an ensemble by training them on random samples of features instead of the entire feature set. Consequently, RSS compels individual learners to not over-focus on variables that appear highly predictive in the training set but fail to be as predictive for points outside that set. Therefore, RSS has gained popularity for high-dimensional problems where the number of variables is significantly larger than the number of training points. Hence, this study employed the RSS model hybridized with DNN to exploit benefits of both techniques while predicting evaporation.

#### M5 pruned

Quinlan^58^ introduced the M5 algorithm, which was further reconstructed to develop the M5 pruned model tree. This integrates the traditional DT with the linear regression function. Wang and Witten^59^ described the four steps in the M5 pruned algorithm; (1) input space splitting, (2) linear regression model development, (3) pruning procedure, and (4) smoothing process. Besides, this algorithm has shown robustness due to its greater efficiency while dealing with missing data problems. Since M5 pruned can efficiently handle and process large datasets to ensure reduced errors in the output, it has been considered for analyzing and predicting the evaporation in the current study area.

The present study acquired data on the splitting measures for the M5 pruned model tree considering the error calculated at each node (linear regression functions are assigned on terminal nodes). The class values’ standard deviation is used to analyze the error at each node. The attribute at each node is tested to select a particular attribute for splitting. This selection is majorly driven by determining the attribute that maximizes the expected error reduction, which can be obtained by standard deviation reduction, as shown in Eq. 8.

8 $$\:SDR=SD\left(A\right){\sum\:}_{1}^{i}\frac{\left|{A}_{i}\right|}{\left|A\right|}SD\left({A}_{i}\right)\:\:\:\:\:\:$$.

where $\:A$ represents the set of instances that attain the node; $\:{\text{A}}_{\text{I}}$ represents the subset of illustrations that have the $\:{\text{i}}^{\text{t}\text{h}}$ product of the possible set, and $\:SD$ represents the standard deviation.

#### Random forest (RF)

The RF model yields comparatively higher performance while constructing ensembles. The learning algorithms of DTs rely on classification and regression tree. Considering architecture, RF comprises sets of DTs, wherein space occupied by each variable is further subdivided into smaller and smaller sub-spaces, achieving uniform space for each data/variable. The structure of DT is employed for this classification pattern such that two sub-branches originating from a branching point are recognized as a node. Furthermore, the root is identified as the first node in the tree structure, while the leaf is identified as the last node^60^. Each of these trees develops with a self-serving sample of the original data. To achieve the best division, a variable, randomly selecting the ‘m’ number of variables, is searched^61^. In the selection process, data discrepancy, in terms of data similarity, is estimated considering their assignment in the final subspaces (leaves of the same kind) instead of using the distance functions method. Equation 9 quantifies the data similarity between data $\:a$ and $\:b$ [$\:s\left(a,\:b\right)$], where it showcases the proportion of the number of times the data provided [$\:d(a,\:b)$] are located in the same final subspaces. In order to convert the similar matrix (data similarity issue) into a non-similar matrix, a forest similarity matrix, which is characteristically random, symmetric, and positive, is employed as per Eq. 9. In general, since the values of the variables do not influence the classification tree formation process, the deficiency of similarity of the RF is applied to the various variable categories. Once the predefined stop condition is attained, the reiteration of the splitting procedure is stopped^62^.

9 $$\:d\left(a,\:b\right)=\sqrt{1}-s\left(a,\:b\right)\:\:\:$$.

As far as the advantages of RF models are concerned, past studies indicated their high efficiency in learning target class samples retrieved from training data alongside unique characteristics from unclassified data^6,35,63^. As a result, RF develops better prediction ability as a hybrid method and overcomes the limitations of non-supervised classifier methods. Therefore, the present study hybridized RF with DNN for modeling pan evaporation.

### Model performance evaluation indicators

In this research, five statistical metrics are applied to evaluate the performance of the developed models; determination coefficient (R^2^), root mean square error (RMSE), mean absolute error (MAE), Nash–Sutcliffe efficiency (NSE), and percentage bias (PBIAS). These metrics are listed as follows: (1) R^2^ [unitless], which evaluates the linear relationship between predicted and observed Ep values; (2) RMSE [mm/day], which measures the mean error of all estimates; (3) MAE [mm/day], which is commonly used to compute model error or residual; (4) NSE [unitless], which is a metric for determining the accuracy of a model; and (5) PBIAS [%], which quantifies the average tendency of the predicted values to be either overestimated or underestimated compared to the observed values.

Lower RMSE, MAE, and PBIAS values approaching 0 indicate better performance accuracy. Higher R^2^ and NSE values, on the other hand, imply a greater degree of agreement between estimated and observed values. The five error quantification measures are defined as per Eqs. 10–14, respectively. The readers may refer to already published literature to see their applications^64–72^.

10 $$\:{R}^{2}=\frac{{\left[\sum\:_{i=1}^{n}\left({x}_{i}-\stackrel{-}{x}\right)\left({y}_{i}-\stackrel{-}{y}\right)\right]}^{2}}{{{s}_{x}}^{2}{{s}_{y}}^{2}}$$

11 $$\:RMSE=\:\sqrt{\sum\:_{i=1}^{n}\frac{{({x}_{i}-{y}_{i})}^{2}}{n}}$$

12 $$\:MAE=\frac{1}{n}\sum\:_{i=1}^{n}\left|{x}_{i}-{y}_{i}\right|$$

13 $$\:NSE=1-\:\frac{\sum\:_{\text{i}=1}^{\text{n}}{\left({\text{x}}_{\text{i}}-{\text{y}}_{\text{i}}\right)}^{2}}{\sum\:_{\text{i}=1}^{\text{n}}{\left({\text{x}}_{\text{i}}-\stackrel{-}{\text{x}}\right)}^{2}}$$

14 $$\:PBIAS=\:\frac{\sum\:_{\text{i}=1}^{\text{n}}\left({\text{x}}_{\text{i}}-{\text{y}}_{\text{i}}\right)}{\sum\:_{\text{i}=1}^{\text{n}}\left({\text{x}}_{\text{i}}\right)}\times\:100$$.

where $\:{\text{x}}_{\text{i}}$ and $\:{\text{y}}_{\text{i}}$ are observed and predicted data, respectively; $\:\stackrel{-}{x}$ and $\:\stackrel{-}{y}$ are the mean observed and predicted data, respectively; $\:{{s}_{x}}^{2}$ and $\:{{s}_{y}}^{2}$ are observed and predicted variances, respectively.

## Methodology

**Fig. 2:**
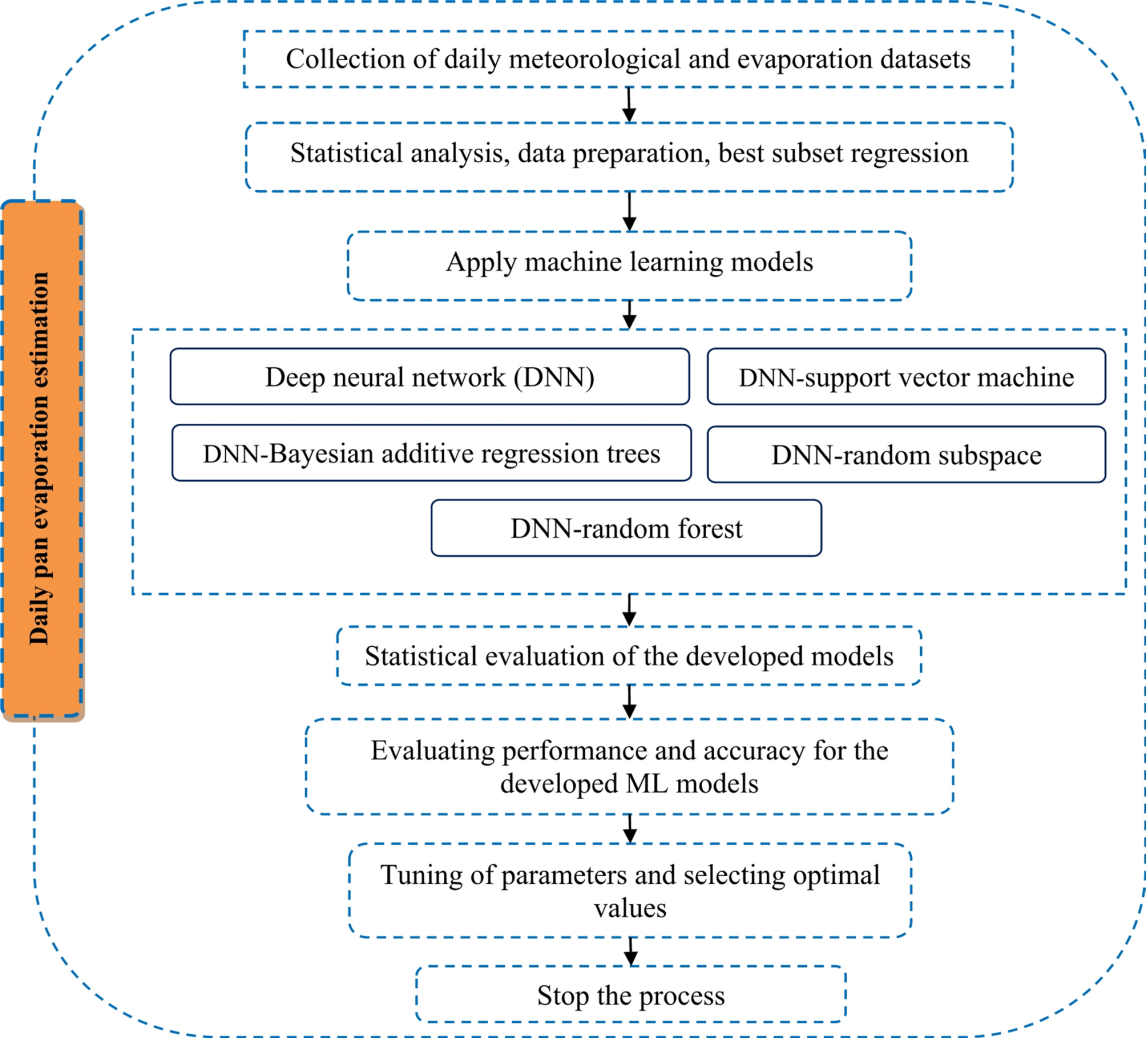
Flowchart of daily pan evaporation estimation methodology in the study area.

The main objective of this research is to estimate evaporation using several ML models in semi-arid regions. The flowchart of the research study is illustrated in Fig. 2, outlining the key steps involved in the methodology. The process begins with collecting daily meteorological datasets, including temperature, relative humidity, wind speed, insolation, and daily evaporation data. Using regression analysis and sensitivity tests, these datasets are then subjected to statistical analysis and data preparation to identify the best subset of input variables. This step is crucial for determining the optimal combination of meteorological factors influencing evaporation.

Following selecting the best input combination, the data is split into training and testing sets with a ratio of 75:25. This division is essential for training the models and evaluating their performance. The stacking process used in this study involves two layers: the first layer consists of multiple base models (DNN, SVM, BART, RSS, M5 pruned, and RF), and the second layer is a meta-model that combines the predictions from the base models. This approach allows for integrating diverse models, leveraging their strengths to improve overall performance. The training set is used to train the base models, and their outputs are then used as inputs for the meta-model, which is trained to optimize the final predictions.

The architecture of the individual models is designed to capitalize on their respective strengths. The DNN model used in this study consists of multiple hidden layers with a rectified linear unit activation function. The input layer receives the meteorological variables (T_min_, T_max_, RH_min_, RH_max_, insolation, and wind speed), and the output layer provides the predicted evaporation values. The number of neurons in each hidden layer was optimized through a grid search to minimize overfitting and improve model performance. SVM is a supervised max-margin model that maps the original finite-dimensional space into a higher-dimensional space to improve separability. It uses kernel functions, such as the Gaussian kernel, to compute dot products efficiently. The effectiveness of SVM depends on the selection of kernel parameters and the soft margin parameter, which are optimized using cross-validation.

BART is a flexible model for regression problems, combining multiple regression trees to model complex relationships between variables. This study uses BART as a base model in the stacking process to leverage its ability to handle non-linear interactions. RSS is an ensemble method that selects random subsets of features to train multiple models, reducing overfitting by averaging predictions across different feature spaces. The M5 model tree is a decision tree-based model incorporating linear regression functions at each leaf node, providing a robust framework for predicting evaporation. RF is an ensemble model that combines multiple decision trees to improve prediction accuracy, with each tree trained on a random subset of features and samples.

The hybrid models integrate the strengths of these base models. The DNN-SVM model combines the feature extraction capabilities of DNNs with the robust classification power of SVMs. The DNN-BART model integrates the non-linear modeling capabilities of BART with the DNNs ability to extract complex features. The DNN-RSS model leverages the feature extraction of DNNs, and the ensemble diversity provided by RSS. The DNN-M5 pruned model combines the strengths of DNNs with the linear regression capabilities of the M5 model tree, enhancing the model’s ability to handle continuous data. Finally, the DNN-RF model integrates the feature extraction of DNNs with the ensemble averaging of RF, providing robust and accurate predictions. For additional understanding of the methodological steps and ML architectures, readers may refer to relevant studies such as those by Samantaray and Ghose^73–75^, Masood et al.^76^ and Elbeltagi et al.^77^.

After developing and running these models, their performance is evaluated using metrics such as MAE, RMSE, R², NSE, and PBIAS. This evaluation stage is critical for selecting the best evaporation forecasting model. A Taylor diagram is also used to supplement the performance assessment measures, comprehensively verifying the optimum prediction model. The process concludes with selecting the most accurate model based on these evaluations, which can be used for practical applications in water resource management.

## Results

### Sensitivity analysis and best subset regression

The following sub-sections cover the best subset regression and sensitivity analyses for determining the optimum input combination for Ep prediction.

#### Input selection using the best subset model

Identifying the correct input parameters is crucial in achieving the greatest model performance. Eight meteorological variables (T_min_, T_max_, T_mean_, RH_min_, RH_max_, RH_mean_, insolation, and wind speed) are employed in this study to determine the best input combination based on several performance indicators, as shown in Table 3. Overfitting is more likely when there are many input variables and a low correlation between input and output^23,78,79^a. For daily Ep prediction, seven statistical criteria were used to identify the optimal input combination: MSE, R^2^modified R^2^Mallows’ Cp, Amemiya prediction criterion (PC), Schwarz Bayesian criterion (SBC), and Akaike information criterion (AIC). Table 3 shows that the bold row is the optimal input combination (T_min_, T_max_, RH_min_, RH_max_, insolation, and wind speed) since it has the lowest Mallows’ Cp (6.18) and Amemiya’s PC (0.34) values, as well as the greatest R^2^ (0.66) and modified R^2^ (0.66) values of all input situations. This scenario is followed by the 6th input combination (T_min_, T_max_, T_mean_, RH_mean_, insolation, and wind speed). Whereas considering a single variable as input (T_mean_) results in the lowest R^2^ and adjusted R^2^ (0.59) and the highest values of other statistical criteria, representing the worst input variable in estimating daily Ep. Following normalization, the entire dataset is divided into two groups, with 75% of the dataset used for training and the remaining 25% used for testing and validating the models.

**Table 3:** The best subset regression analysis for identifying the optimum input combinations for ep prediction.

ID	Variables	MSE	*R* ^2^	Adjusted *R*^2^	Mallows’ Cp	Akaike’s AIC	Schwarz’s SBC	Amemiya’s PC
1	T_mean_	11.87	0.59	0.59	815.78	9942.92	9955.51	0.41
2	T_min_/Insolation	10.55	0.64	0.64	280.75	9471.30	9490.19	0.36
3	T_min_/RH_mean_/Insolation	9.93	0.66	0.66	27.843	9227.33	9252.53	0.34
4	T_min_/T_max_/RH_mean_/Insolation	9.88	0.66	0.66	10.31	9209.85	9241.34	0.34
5	T_min_/T_max_/RH_mean_/Insolation/Wind speed	9.87	0.66	0.66	4.25	9203.78	9241.57	0.34
6	T_min_/T_max_/T_mean_/RH_mean_/Insolation/Wind speed	9.87	0.66	0.66	6.07	9205.60	9249.69	0.34
7	T_min_/T_max_/RH_min_/RH_max_/Insolation/Wind speed	9.87	0.68	0.68	6.18	9205.71	9249.80	0.34
8	T_min_/T_max_/T_mean_/RH_min_/RH_max_/Insolation/Wind speed	9.87	0.66	0.66	8.00	9207.53	9257.92	0.34

#### Sensitivity analysis

**Fig. 3:**
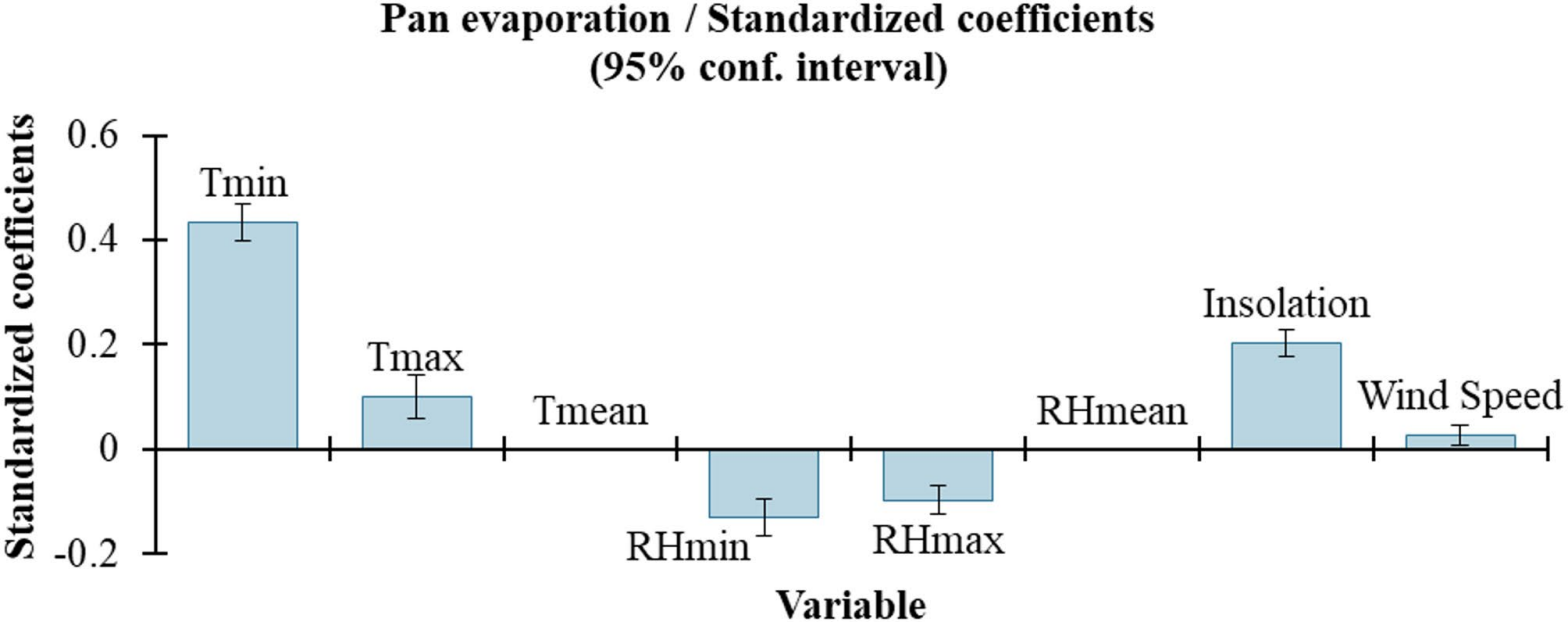
Standardized coefficients of the input variables for sensitivity analysis.

The combinations of input variables significantly influence the performance of data-driven models. Some variables contribute positively to model accuracy, while others may have a negative impact. To identify the most influential input parameters for predicting daily Ep, we conducted a regression analysis. The results in Table 4; Fig. 3 highlight the importance of T_min_, T_max_, RH_min_, RH_max_, insolation, and wind speed.

The statistical significance of each variable was assessed using t-tests, with the p-values indicating the probability of observing the test statistic under the null hypothesis that the coefficient is zero. A p-value less than 0.05 indicates that the variable has a statistically significant effect on the model. T_min_, with a coefficient of 0.433 and a p-value of less than 0.0001 (t = 23.220), is highly significant, suggesting that T_min_ has a strong positive influence on the prediction of Ep. T_max_ also positively affects Ep predictions, though to a lesser extent than T_min_, with a coefficient of 0.101 and a p-value of less than 0.0001 (t = 4.731).

In contrast, RH_min_ and RH_max_ have negative coefficients, negatively impacting Ep predictions. RH_min_ has a coefficient of − 0.130 with a p-value of less than 0.0001 (t = − 7.142), while RH_max_ has a coefficient of − 0.097 with a p-value of less than 0.0001 (t = − 6.644). Insolation, with a coefficient of 0.202 and a p-value of less than 0.0001 (t = 16.291), is a highly significant positive predictor of Ep. Wind speed also positively affects Ep, though to a lesser extent, with a coefficient of 0.027 and a p-value of 0.005 (t = 2.842). T_mean_ and RH_mean_ were found to have no significant impact, as indicated by their coefficients of 0.000 and associated p-values.

This analysis demonstrates that T_min_, T_max_, RH_min_, RH_max_, insolation, and wind speed are the most influential input parameters for predicting daily Ep, with T_min_ and insolation being particularly significant.

**Table 4:** The regression analysis for identifying the most effective parameters for ep estimation.

Source	Value	Standard error	t	Pr > |t|	Lower bound (95%)	Upper bound (95%)
T_min_	0.433	0.019	23.220	< 0.0001	0.397	0.470
T_max_	0.101	0.021	4.731	< 0.0001	0.059	0.143
T_mean_	0.000	0.000				
RH_min_	− 0.130	0.018	− 7.142	< 0.0001	− 0.165	− 0.094
RH_max_	− 0.097	0.015	− 6.644	< 0.0001	− 0.126	− 0.068
RH_mean_	0.000	0.000				
Insolation	0.202	0.012	16.291	< 0.0001	0.178	0.227
Wind speed	0.027	0.009	2.842	0.005	0.008	0.045

### Implementation of machine learning models for daily ep estimation

The estimation of Ep at Sidi Yakoub station in the Wadi Sly basin is carried out using DNN and its hybrids (i.e., SVM, BART, RSS, M5 pruned, and RF). The parameters of the ML models used for pan evaporation modeling are depicted in Table 5. Comparative performances are evaluated using statistical performance indicators such as R^2^ NSE, RMSE, and MAE. The model with the highest values of R^2^ and NSE close to one as well as the lowest values of RMSE and MAE approaching zero is considered the best predictive model for Ep.

The values of R^2^ NSE, RMSE, and MAE for the predictive models during the training and testing periods are presented in Table 6. As depicted in Table 6, RF can enhance the performance of DNN and shows the best statistical performance criteria during the training period (RMSE = 1.000, MAE = 0.958, and NSE = 0.922). However, during the testing span, the DNN-SVM model shows superiority over the other models and has statistical performance criteria values of RMSE = 3.000, MAE = 2.127, and NSE = 0.649. Therefore, the DNN-SVM is selected as the optimum predictive model because it is associated with the best statistical criteria (i.e., minimum RMSE and MAE as well as maximum NSE) in the testing stage.

**Table 5:** Parameters of the machine learning algorithms used for pan evaporation modeling.

Model name	Description of parameters
DNN	Batch size-100, Learning rate = 0.3, Momentum = 0.2, Auto build = True, Nominal to Binary = True, Normalize Attributes = True, Normalize Numeric Class = True, Debug = False, Decay = False
AR	Batch size-100, Classifier = Bagging, shrinkage = 1, number of iterations = 30
SVM	Batch size-100, C = 0.1, kernel used = polykernel
RF	Batch size-100, bag Size percent = 100, max depth = 0, numbers of executions slots = 1, number of iterations = 100, random seed = 1
M5 pruned	Batch size-100, Minimum number of instances = 4
RSS	Batch size-100, Classifier = REPTree, random seed-1, subspace size = 0.5, numbers of executions slots = 1, number of iterations = 10

**Table 6:** Performance criteria for the models during the training and testing phases.

Models	Training					
	*R* ^2^	RMSE	NRMSE	MAE	NSE	PBIAS
DNN	0.797	5.000	0.656	4.742	0.002	− 60.31
DNN-SVM	0.795	2.000	0.262	1.630	0.750	0.421
DNN-BART	0.789	2.000	0.262	1.682	0.788	0.388
DNN-RSS	0.809	2.000	0.262	1.583	0.809	− 0.157
DNN-M5 pruned	0.795	2.000	0.262	1.645	0.794	0.419
DNN-RF	0.924	1.000	0.131	0.958	0.922	1.010
Models	Testing					
DNN	0.668	5.000	0.654	4.870	0.061	− 58.099
DNN-SVM	0.651	3.000	0.393	2.127	0.649	3.540
DNN-BART	0.630	3.000	0.393	2.220	0.630	0.761
DNN-RSS	0.631	3.000	0.393	2.165	0.628	1.744
DNN-M5 pruned	0.635	3.000	0.393	2.166	0.633	2.309
DNN-RF	0.638	3.000	0.393	2.155	0.638	0.411

**Fig. 4:**
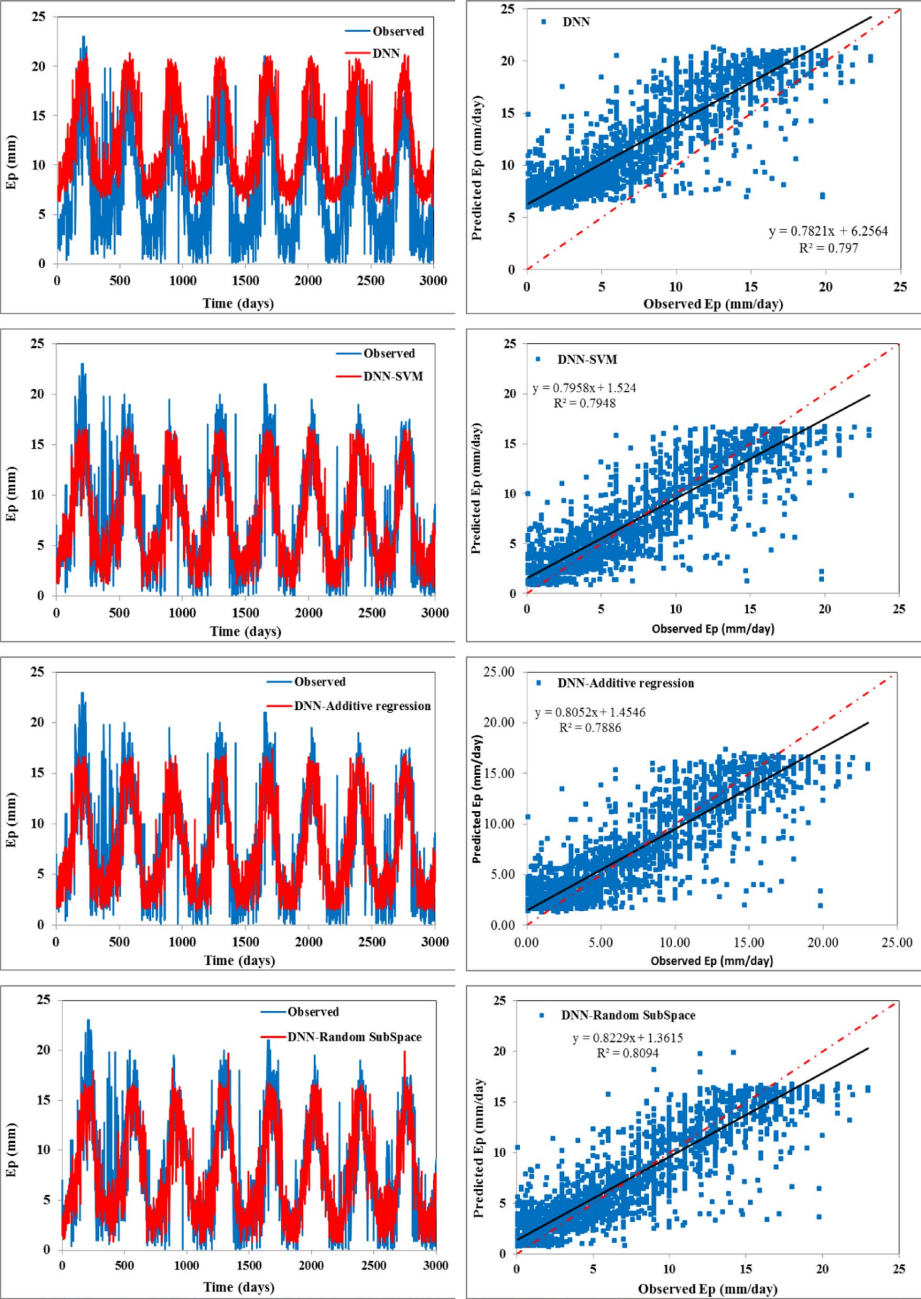

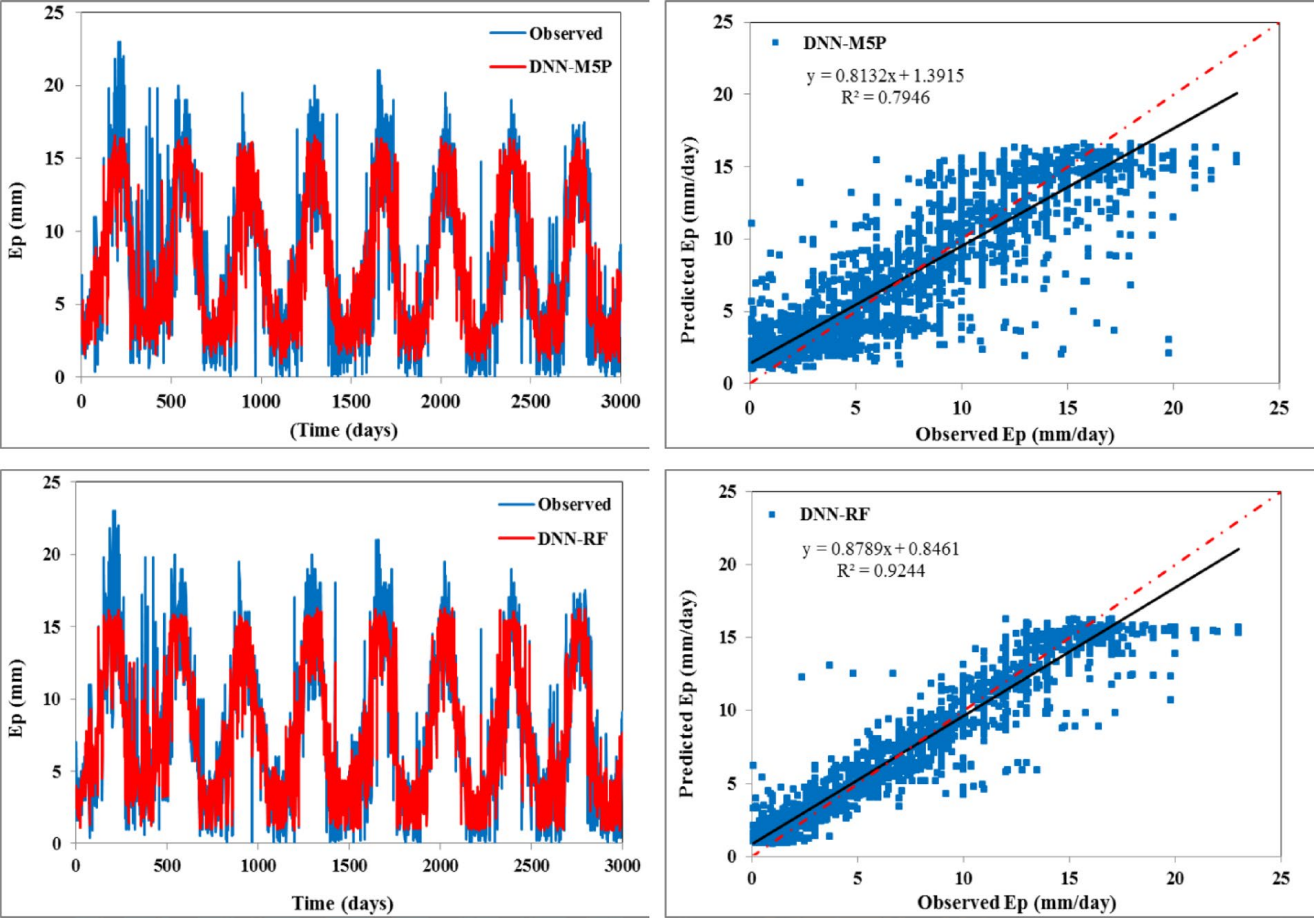
Temporal variation (left) and scatter plot (right) of observed vs. predicted daily Ep during the training span.

**Fig. 5:**
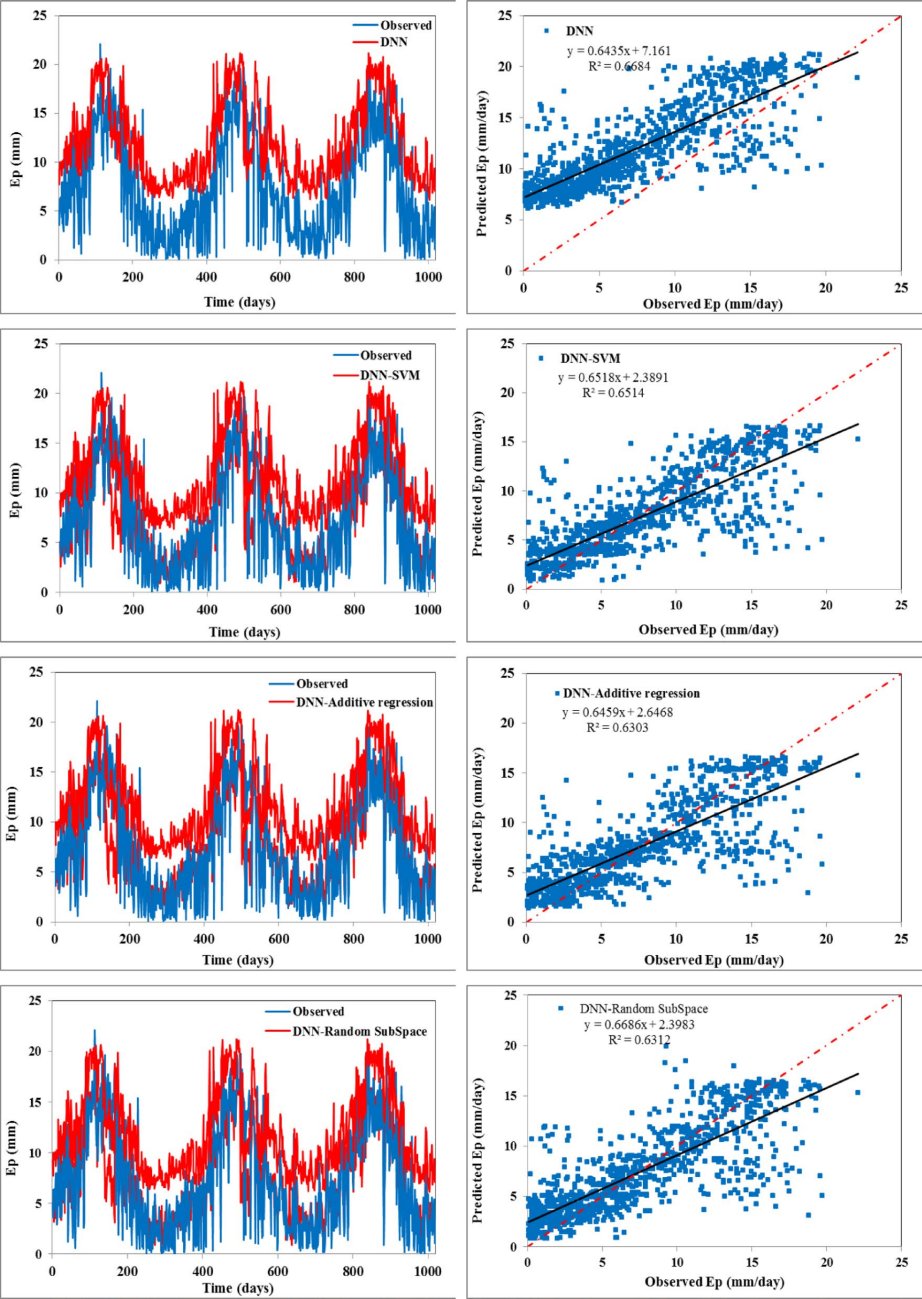

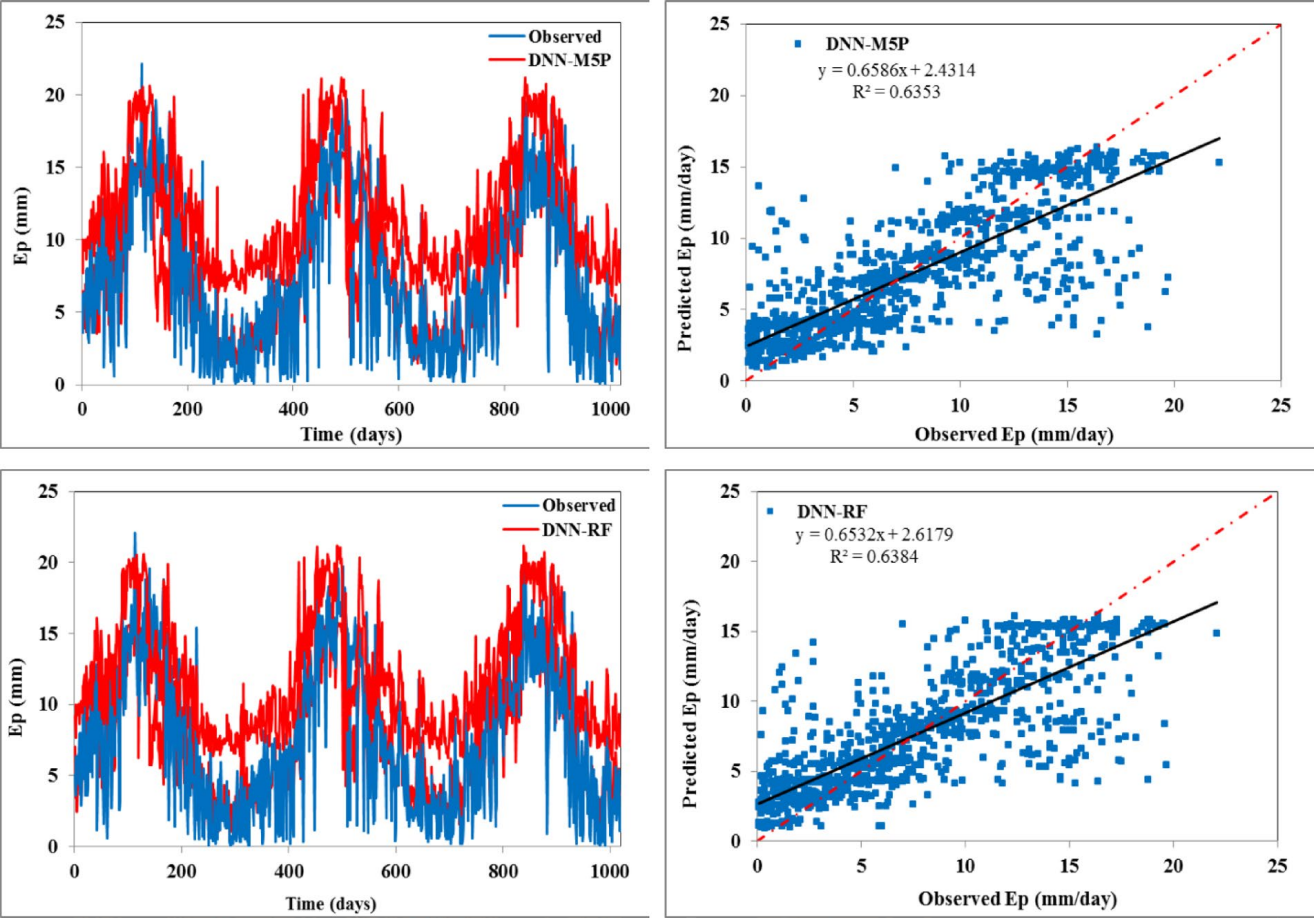
Temporal variation (left) and scatter plot (right) of observed vs. predicted daily Ep during the testing span.

The scatter plots (right side) and temporal variation (left side) between predicted and observed daily evaporation values are plotted in Figs. 4 and 5 for the training and testing periods, respectively. In scatter plots, the regression line provides the R^2^ value as 0.668 for the DNN model, 0.651 for the DNN-SVM model, 0.630 for the DNN-BART, 0.631 for the DNN-RSS model, 0.635 for the DNN-M5 pruned model, and 0.638 for the DNN-RF model during the testing stage, respectively. The fitted regression line and the perfect line of fit (1:1) are almost identical for all the hybrid models except the standalone DNN model. This reveals that all the constructed hybrid models can enhance the performance of DNN.

**Fig. 6:**
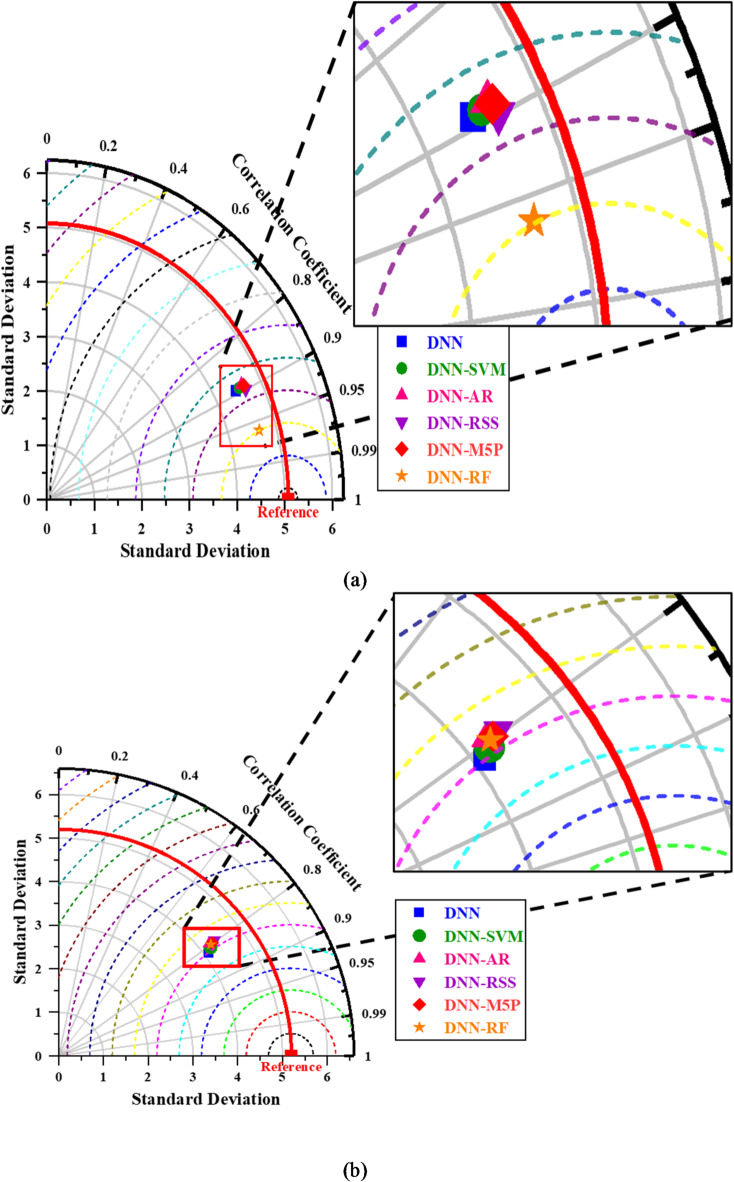
Taylor diagrams of the prediction models during (**a**) training and (**b**) testing phases.

Besides, the Taylor diagram is used to visually assess the efficiency of data-driven DNN and hybrid models. The fundamental advantage of this graphical approach is that it summarizes three important statistical criteria in a single chart: RMSE, correlation coefficient, and standard deviation^23,80^. Furthermore, it displays the model’s correctness and realism, when compared to the observable parameters. The standard deviation stands for the number of average measurements that deviate from one another^81^. As a result, high precision is indicated by the relative value of the standard deviation predicted to the standard deviation actual. In contrast, the value of the standard deviation predicted compared to the standard deviation actual denotes inferior accuracy. As shown in Fig. 6, the Taylor diagram is used to conduct a comparative analysis of models in this study. For the training phase, the DNN-RF model is closer to the observed point and shows superiority during the training period. For the testing phase, all the hybrid models compete; however, the DNN-SVM model provides a slightly better result than other models as it has the highest correlation coefficient and lowest RMSE and standard deviation values.

## Discussion

According to the subset regression analysis results, the best input combination for Ep prediction included T_min_, T_max_, RH_min_, RH_max_, insolation, and wind speed. Previous studies showed that these factors physically impacted Ep^79,82^. The best subset combination was used to predict the daily Ep by constructing DNN, DNN-SVM, DNN-BART, DNN-RSS, DNN-M5 pruned, and DNN-RF models. The results showed that DNN-RF performed better during the training span; however, DNN-SVM showed superiority during the testing period. Therefore, the DNN-SVM model could be utilized as a prediction tool for daily evaporation in the selected station under semi-arid conditions. The applications of the model in various contexts may only be conceivable after they have been calibrated with new data. Besides, combining DNN and SVM models integrates the feature extraction capabilities of DNNs and the robust classification power of SVMs. For instance, Huynh et al.^83^ proposed a hybrid model that utilizes DNNs for automatic feature extraction from high-dimensional gene expression data, followed by SVM for classification, resulting in improved accuracy over standalone models. Similarly, Díaz-Vico et al.^84^ developed deep SVM models by integrating the non-linear feature extraction capabilities of DNN while incorporating the loss functions of SVM. This approach achieved comparable performance to standard SVM but with the added benefit of improved scalability for larger datasets. Along these lines, some of the other research for different applications (beyond hydrology) include Ahmad et al.^85^, Ma et al.^86^, Jo et al.^87^, Elbeltagi et al.^88^ and Prasunna et al.^89^. These findings suggest that a DNN-SVM hybrid model could effectively combine the strengths of both approaches, potentially leading to superior performance in daily evaporation prediction as well under semi-arid conditions.

Other recent research^3,79,90,91^ conducted on different continents of the world corroborated the findings of the current study. Lin et al.^91^ compared two ML algorithms for estimating daily evaporation values (SVM and backpropagation network). The results showed that SVM has a high capacity to predict daily Ep values and could be a viable alternative for Ep estimates. Malik et al.^3^ compared the accuracy of five ML algorithms (i.e., multivariate adaptive regression spline, SVM, multi-gene genetic programming, multiple model-ANN, and M5 model tree) in predicting the monthly Ep in India. The authors found that the multi-gene genetic programming and multiple model-ANN algorithms outperformed the SVM and multivariate adaptive regression spline methods and the M5 model tree approach in terms of prediction performance, as evidenced by their high RMSE values. Kushwaha et al.^79^ tested four ML algorithms (SVM, RSS, random tree, and reduced error pruning tree) in Northern India under various climate circumstances. According to the study, SVM outperformed other applied algorithms because it had high R and Willmott index values and low MAE and RMSE values. Chen et al.^90^ studied the efficacy of SVM for monthly Ep prediction at six distinct sites along the Yangtze River in China. The SVM was superior to standard approaches for estimating Ep in the study. The current study also shows that the DNN-SVM hybrid ML method predicts daily Ep more accurately than other algorithms. Overall, findings show hybrid models are more predictive in real-world scenarios and may be used effectively in watersheds with limited data. These models could forecast a wide range of hydrological and water resource phenomena besides Ep.

Despite its contributions, this study has certain limitations. Since the primary objective was to evaluate the comparative performance of standalone and hybrid DNN models, alternative deep learning architectures such as convolutional neural network, gated recurrent units, and other hybrid frameworks were not explored. Future studies could extend this research by incorporating these advanced models to improve prediction accuracy further and assess their generalizability across diverse climatic conditions. Additionally, while the current study explains Ep estimation, multi-step ahead forecasting was not within its scope. Future research should explore long-term Ep prediction using hybrid models integrated with temporal learning mechanisms such as long short-term memory and transformer-based architectures, which have demonstrated superior sequence modeling capabilities (e.g., Roy et al.^92^). Expanding the model to diverse climatic regions will help validate its robustness while integrating remote sensing and satellite data can improve spatial accuracy. Moreover, the study’s findings should be validated across other semi-arid regions worldwide to assess their adaptability under different environmental settings, as suggested by El-Kenawy et al.^93^. Comparative studies across multiple geographical contexts would boost the credibility and applicability of hybrid models in hydrological forecasting. Additionally, uncertainty quantification through Bayesian learning can improve model reliability and refine interpretability. Coupling artificial intelligent (AI) models with climate change projections can predict future evaporation trends under different scenarios. Furthermore, real-time applications such as AI-driven decision support systems and early warning mechanisms can aid water resource management. Evolutionary optimization techniques like genetic algorithms and swarm intelligence can refine model efficiency, while policy-driven studies can assess the socioeconomic impacts of evaporation variability. Integrating hybrid deep learning models with soil moisture indices and large-scale climate predictors such as the El Niño-Southern Oscillation and the Madden-Julian Oscillation could improve evaporation estimation under changing climate scenarios. These advancements will strengthen AI-driven hydrological modeling for sustainable water management in arid and semi-arid regions.

## Conclusions

This study evaluated the effectiveness of standalone and hybrid DNN models in estimating Ep in the semi-arid Sidi Yakoub meteorological station, Wadi Sly basin, Algeria. Hybrid models were constructed by integrating DNN with advanced ML algorithms, including SVM, BART, RSS, M5 pruned, and RF. A comprehensive dataset spanning 2000–2010 was utilized, with 75% designated for training and 25% for testing. Subset regression analysis identified the most influential meteorological variables (i.e., wind speed, sunshine hours, maximum and minimum temperature, and humidity) followed by sensitivity analysis to determine their predictive significance. Performance evaluation using multiple statistical metrics revealed that hybrid models consistently outperformed standalone DNN models. Among them, the DNN-SVM model demonstrated the highest accuracy (R^2^ = 0.651, RMSE = 3.000, MAE = 2.127, NSE = 0.649, and PBIAS = 3.540), highlighting its ability to capture complex nonlinear relationships between meteorological parameters and Ep variations. Notably, models incorporating maximum and minimum temperature and humidity, rather than average values, exhibited superior predictive capabilities, reinforcing the importance of precise meteorological inputs. The findings underscore the potential of DNN-SVM as a robust and reliable predictive tool for Ep estimation in semi-arid environments, with broader applicability across Algeria. The study contributes to improving hydrological modeling and water resource management by providing a data-driven, machine-learning-based framework for evaporation forecasting. Future research could refine model performance by integrating wavelet packet decomposition and complete ensemble empirical mode decomposition, further enhancing predictive accuracy in diverse climatic conditions.

## Electronic supplementary material

Below is the link to the electronic supplementary material.

Supplementary Material 1

## Data Availability

The authors confirm that the data supporting the findings of this study are available within the article.

## Abbreviations

AI: Artificial intelligence
AIC: Akaike information criterion
ANN: Artificial neural network
BART: Bayesian additive regression trees
DNN: Deep neural network
DT: Decision tree
Ep: Pan evaporation
MAE: Mean absolute error
ML: Machine learning
NSE: Nash–Sutcliffe efficiency
P (mm): Precipitation
PBIAS: Percentage bias
PC: Amemiya prediction criterion
R^2^: Determination coefficient
Ra (MJm2): Extra-terrestrial radiation
RF: Random forest
RH_max_ (%): Maximum relative humidity
RH_mean_ (%): Mean relative humidity
RHmin (%): Minimum relative humidity
RMSE: Root mean square error
Rs (MJm2): Solar radiation
RSS: Random subspace
SBC: Schwarz Bayesian criterion
SSH (hours): Sunshine hours
SVM: Support vector machine
T_max_ (℃): Maximum temperature
T_mean_ (℃): Mean temperature
T_min_ (℃): Minimum temperature
VP (hPa): Vapor pressure
WSP (ms^−1^): Wind speed
