# Supplementary figures and images for: Comparative assessment of standalone and hybrid deep neural networks for modeling daily pan evaporation in a semi-arid environment
Source: Sci Rep. 2025 Jun 20;15:20179. doi: 10.1038/s41598-025-05985-z (PMC12181372; doi:10.1038/s41598-025-05985-z)

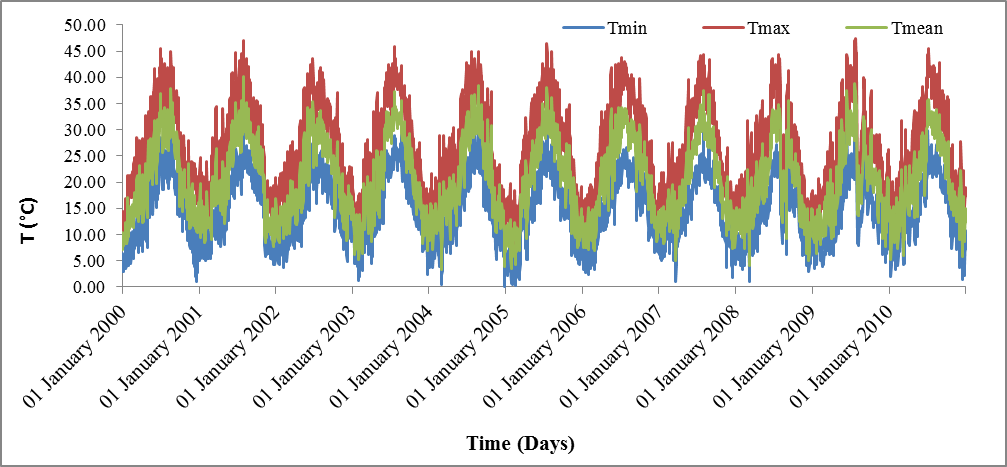


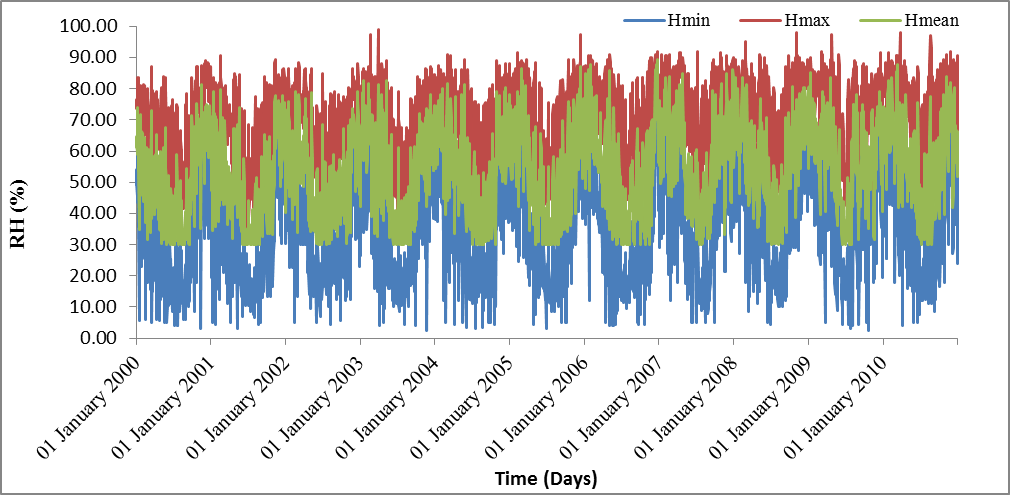


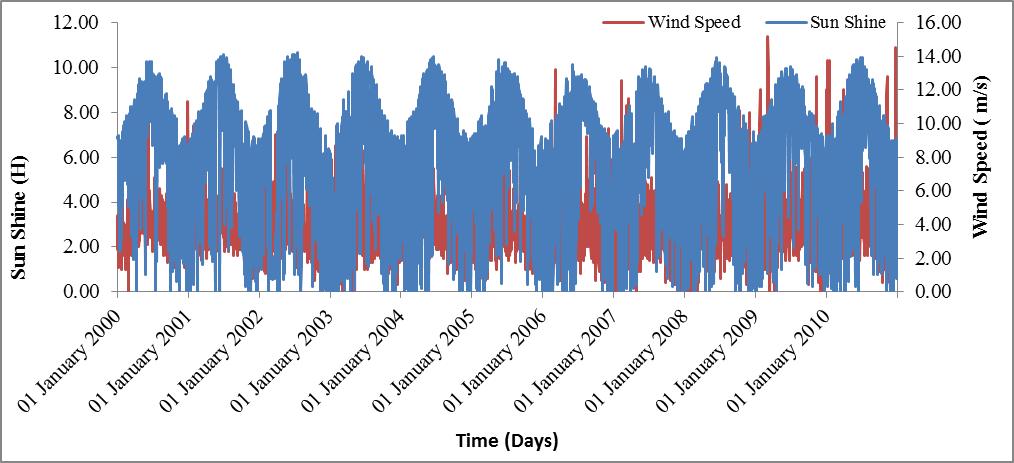


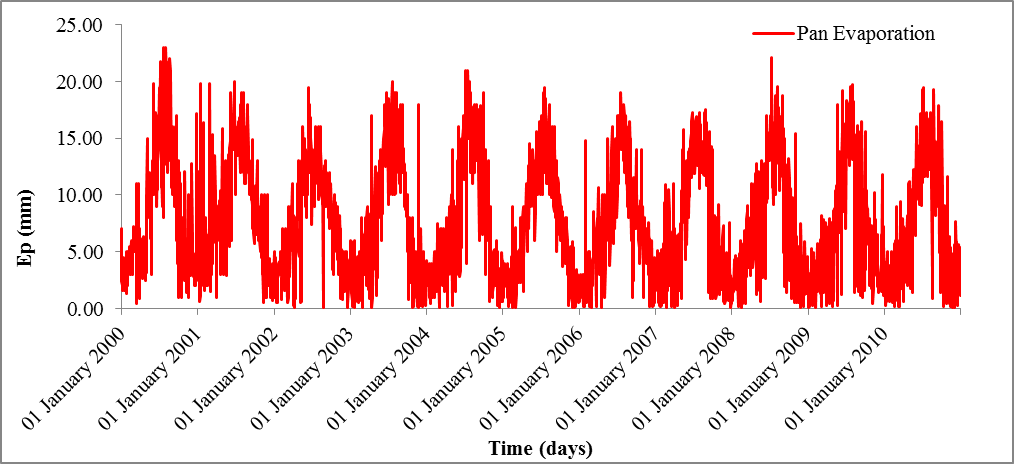


**Figure S1** Daily meteorological data and pan evaporation from January 2000 to December 2010

Supplement: Supplementary file 1 — Supplementary Material 1 [file 41598_2025_5985_MOESM1_ESM.docx]
